# Methyl 3-(cyclo­propyl­meth­oxy)-4-hy­droxy­benzoate

**DOI:** 10.1107/S1600536810026826

**Published:** 2010-07-14

**Authors:** Jing-Jing Hou, Xian-Chao Cheng, Run-Ling Wang, Shu-Qing Wang

**Affiliations:** a School of Pharmacy, Tianjin Medical University, Tianjin 300070, People’s Republic of China

## Abstract

In the title compound, C_12_H_14_O_4_, the dihedral angle between the benzene ring and the cyclo­propyl ring is 60.3 (4)°. In the crystal structure, mol­ecules are linked by inter­molecular O—H⋯O hydrogen bonds into chains running parallel to [101].

## Related literature

For bond-length and angle data for related structures, see: Bradley *et al.* (1992 ▶); Fifer & White (2005 ▶). During the development of PDE4 (phospho­diesterase-4) inhibitors, roflumilast was synthesized as the positive control in the bioactivity screening and the title compound was prepared as an inter­mediate. For the synthesis of roflumilast, see: Bose *et al.* (2005 ▶).
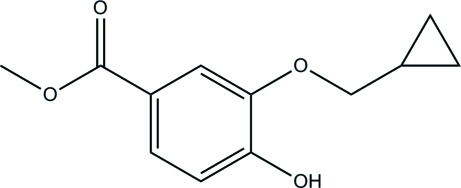


## Experimental

### 

#### Crystal data

C_12_H_14_O_4_
*M* *_r_* = 222.23
Monoclinic, 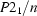

*a* = 9.2326 (18) Å
*b* = 7.4747 (15) Å
*c* = 16.105 (3) Å
β = 102.22 (3)°
*V* = 1086.3 (4) Å^3^
*Z* = 4
Mo *K*α radiation
μ = 0.10 mm^−1^
*T* = 113 K
0.24 × 0.22 × 0.12 mm

#### Data collection

Rigaku Saturn CCD area-detector diffractometer
Absorption correction: multi-scan (*CrystalClear*; Rigaku, 2005 ▶) *T*_min_ = 0.976, *T*_max_ = 0.988
7033 measured reflections
1904 independent reflections
1614 reflections with *I* > 2σ(*I*)
*R*_int_ = 0.031

#### Refinement

*R*[*F* ^2^ > 2σ(*F* ^2^)] = 0.036
*wR*(*F* ^2^) = 0.100
*S* = 1.06
1904 reflections
148 parameters
H-atom parameters constrained
Δρ_max_ = 0.27 e Å^−3^
Δρ_min_ = −0.18 e Å^−3^

### 

Data collection: *CrystalClear* (Rigaku, 2005 ▶); cell refinement: *CrystalClear*; data reduction: *CrystalClear*; program(s) used to solve structure: *SHELXTL* (Sheldrick, 2008 ▶); program(s) used to refine structure: *SHELXTL*; molecular graphics: *SHELXTL*; software used to prepare material for publication: *SHELXTL*.

## Supplementary Material

Crystal structure: contains datablocks I, global. DOI: 10.1107/S1600536810026826/rz2473sup1.cif

Structure factors: contains datablocks I. DOI: 10.1107/S1600536810026826/rz2473Isup2.hkl

Additional supplementary materials:  crystallographic information; 3D view; checkCIF report

## Figures and Tables

**Table 1:** Hydrogen-bond geometry (Å, °)

*D*—H⋯*A*	*D*—H	H⋯*A*	*D*⋯*A*	*D*—H⋯*A*
O3—H3⋯O1^i^	0.84	2.00	2.7808 (14)	153

Symmetry code: (i) 
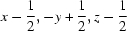
.

**Fig. 1.**
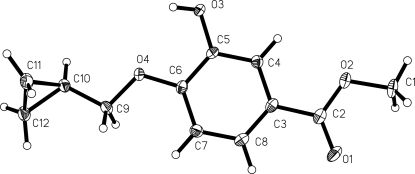
The molecular structure of the title compound, with displacement ellipsoids drawn at the 40% probability level.

## supplementary crystallographic information

### Comment

Roflumilast is an effective phosphodiesterase-4 inhibitor (PDE4 inhibitor), which can be used in the treatment of asthma, inflammation, bronchitis, allergy and other disorders related to immune system, heart and kidney. During the development of our own PDE4 inhibitors, roflumilast was synthesized as the positive control in the bioactivity screening, and the title compound, methyl 3-(cyclopropylmethoxy)-4-hydroxybenzoate, was prepared as an intermediate. The crystallographic analysis of the title compound described herein further confirms the phenolic hydroxyl substituted position of the title compound.

In title compound (Fig. 1), bond lengths and angles are normal and in a good agreement with those reported previously (Bradley *et al.*, 1992; Fifer & White, 2005). Atoms O1—O4/C1—C8 are coplanar, with a maximum displacement of 0.028 (3) Å for atom O4. The dihedral angle between the benzene ring (C3—C8) and cyclopropyl ring (C10—C12) is 60.3 (4)°. In the crystal structure, molecules interact through intermolecular O—H···O hydrogen bonds (Table 1) to form chains running parallel to the [101] direction.

### Experimental

A solution of 3,4-dihydroxymethyl benzoate (1.68 g, 10 mmol) and potassium carbonate (2.76 g, 20 mmol) in acetone (50 ml) was added to a solution of cyclopropylmethyl bromide (1.35 g, 10 mmol) in acetone (50 ml). The reaction mixture was stirred at 40 ° C for 18 h, and then was filtered. The filtrate was evaporated in a rotary evaporator to get the dried crude product. Pure title compound (0.43 g, 18% yield) was obtained by flash column chromatography (Bose *et al.*, 2005). Crystals suitable for X-ray diffraction were obtained through slow evaporation of a solution of the pure title compound in ethyl acetate/n-hexane (1:10 *v*/*v*).

### Refinement

All H atoms were found on difference maps and included in the final cycles of refinement using a riding model, with C—H = 0.95–1.00 Å, O—H = 0.84 Å, and with *U*_iso_(H) = 1.2*U*_eq_(C) for aryl and methylene H atoms and 1.5*U*_eq_(C, O) for the methyl and hydroxy H atoms.

### Crystal data

**Table:**

C_12_H_14_O_4_	*F*(000) = 472
*M**_r_* = 222.23	*D*_x_ = 1.359 Mg m^−^^3^
Monoclinic, *P*2_1_/*n*	Mo *K*α radiation, λ = 0.71073 Å
Hall symbol: -P 2yn	Cell parameters from 3285 reflections
*a* = 9.2326 (18) Å	θ = 2.4–27.9°
*b* = 7.4747 (15) Å	µ = 0.10 mm^−^^1^
*c* = 16.105 (3) Å	*T* = 113 K
β = 102.22 (3)°	Block, colourless
*V* = 1086.3 (4) Å^3^	0.24 × 0.22 × 0.12 mm
*Z* = 4	

### Data collection

**Table:**

Rigaku Saturn CCD area-detector diffractometer	1904 independent reflections
Radiation source: rotating anode	1614 reflections with *I* > 2σ(*I*)
confocal	*R*_int_ = 0.031
Detector resolution: 7.31 pixels mm^-1^	θ_max_ = 25.0°, θ_min_ = 2.4°
ω and φ scans	*h* = −10→10
Absorption correction: multi-scan (*CrystalClear*; Rigaku, 2005)	*k* = −8→8
*T*_min_ = 0.976, *T*_max_ = 0.988	*l* = −14→19
7033 measured reflections	

### Refinement

**Table:**

Refinement on *F*^2^	Secondary atom site location: difference Fourier map
Least-squares matrix: full	Hydrogen site location: inferred from neighbouring sites
*R*[*F*^2^ > 2σ(*F*^2^)] = 0.036	H-atom parameters constrained
*wR*(*F*^2^) = 0.100	*w* = 1/[σ^2^(*F*_o_^2^) + (0.070*P*)^2^] where *P* = (*F*_o_^2^ + 2*F*_c_^2^)/3
*S* = 1.06	(Δ/σ)_max_ = 0.001
1904 reflections	Δρ_max_ = 0.27 e Å^−^^3^
148 parameters	Δρ_min_ = −0.18 e Å^−^^3^
0 restraints	Extinction correction: *SHELXTL* (Sheldrick, 2008), Fc^*^=kFc[1+0.001xFc^2^λ^3^/sin(2θ)]^-1/4^
Primary atom site location: structure-invariant direct methods	Extinction coefficient: 0.137 (12)

### Special details

**Table:**

Geometry. All e.s.d.'s (except the e.s.d. in the dihedral angle between two l.s. planes) are estimated using the full covariance matrix. The cell e.s.d.'s are taken into account individually in the estimation of e.s.d.'s in distances, angles and torsion angles; correlations between e.s.d.'s in cell parameters are only used when they are defined by crystal symmetry. An approximate (isotropic) treatment of cell e.s.d.'s is used for estimating e.s.d.'s involving l.s. planes.
Refinement. Refinement of *F*^2^ against ALL reflections. The weighted *R*-factor *wR* and goodness of fit *S* are based on *F*^2^, conventional *R*-factors *R* are based on *F*, with *F* set to zero for negative *F*^2^. The threshold expression of *F*^2^ > σ(*F*^2^) is used only for calculating *R*-factors(gt) *etc*. and is not relevant to the choice of reflections for refinement. *R*-factors based on *F*^2^ are statistically about twice as large as those based on *F*, and *R*- factors based on ALL data will be even larger.

### Fractional atomic coordinates and isotropic or equivalent isotropic displacement parameters (Å^2^)

**Table:**

	*x*	*y*	*z*	*U*_iso_*/*U*_eq_	
O1	1.20469 (11)	0.31399 (12)	0.41048 (5)	0.0325 (3)	
O2	1.30178 (9)	0.17490 (11)	0.31110 (5)	0.0271 (3)	
O3	0.89725 (9)	0.09184 (11)	0.06138 (5)	0.0239 (3)	
H3	0.8217	0.1289	0.0271	0.036*	
O4	0.65026 (9)	0.22515 (10)	0.10058 (5)	0.0201 (3)	
C1	1.44532 (14)	0.17415 (17)	0.36957 (8)	0.0298 (3)	
H1A	1.4785	0.2976	0.3822	0.045*	
H1B	1.5174	0.1108	0.3436	0.045*	
H1C	1.4368	0.1136	0.4223	0.045*	
C2	1.19019 (14)	0.24965 (14)	0.33996 (8)	0.0216 (3)	
C3	1.04746 (14)	0.24370 (13)	0.27724 (7)	0.0192 (3)	
C4	1.03704 (13)	0.16800 (14)	0.19666 (7)	0.0186 (3)	
H4	1.1222	0.1169	0.1817	0.022*	
C5	0.90327 (13)	0.16755 (13)	0.13899 (7)	0.0170 (3)	
C6	0.77661 (13)	0.23945 (13)	0.16152 (7)	0.0175 (3)	
C7	0.78714 (14)	0.31575 (15)	0.24153 (7)	0.0211 (3)	
H7	0.7020	0.3661	0.2568	0.025*	
C8	0.92302 (14)	0.31775 (15)	0.29883 (8)	0.0217 (3)	
H8	0.9305	0.3704	0.3533	0.026*	
C9	0.51186 (13)	0.27000 (15)	0.12305 (7)	0.0215 (3)	
H9A	0.5102	0.3987	0.1374	0.026*	
H9B	0.4996	0.1998	0.1732	0.026*	
C10	0.38989 (13)	0.22846 (15)	0.04938 (7)	0.0213 (3)	
H10	0.3932	0.1075	0.0233	0.026*	
C11	0.32129 (13)	0.37525 (17)	−0.00999 (8)	0.0274 (3)	
H11A	0.3624	0.4974	0.0009	0.033*	
H11B	0.2860	0.3447	−0.0707	0.033*	
C12	0.23882 (13)	0.29816 (16)	0.05244 (8)	0.0241 (3)	
H12A	0.1526	0.2203	0.0301	0.029*	
H12B	0.2291	0.3730	0.1017	0.029*	

### Atomic displacement parameters (Å^2^)

**Table:**

	*U*^11^	*U*^22^	*U*^33^	*U*^12^	*U*^13^	*U*^23^
O1	0.0332 (6)	0.0402 (5)	0.0194 (5)	−0.0017 (4)	−0.0049 (4)	−0.0076 (4)
O2	0.0191 (5)	0.0353 (5)	0.0232 (5)	−0.0006 (3)	−0.0039 (4)	−0.0021 (4)
O3	0.0193 (5)	0.0356 (5)	0.0146 (4)	0.0066 (3)	−0.0014 (3)	−0.0044 (4)
O4	0.0138 (5)	0.0270 (5)	0.0183 (4)	0.0017 (3)	0.0006 (3)	−0.0009 (3)
C1	0.0184 (7)	0.0340 (7)	0.0312 (7)	−0.0028 (5)	−0.0080 (6)	0.0015 (6)
C2	0.0234 (7)	0.0186 (6)	0.0203 (6)	−0.0035 (4)	−0.0006 (5)	0.0027 (5)
C3	0.0224 (7)	0.0165 (6)	0.0166 (6)	−0.0030 (4)	−0.0003 (5)	0.0020 (4)
C4	0.0177 (6)	0.0190 (6)	0.0189 (6)	0.0004 (4)	0.0034 (5)	0.0012 (5)
C5	0.0205 (6)	0.0164 (6)	0.0132 (6)	−0.0008 (4)	0.0016 (5)	0.0002 (4)
C6	0.0178 (6)	0.0163 (6)	0.0172 (6)	−0.0011 (4)	0.0008 (5)	0.0031 (4)
C7	0.0208 (7)	0.0206 (6)	0.0223 (6)	0.0002 (5)	0.0058 (5)	−0.0016 (5)
C8	0.0272 (7)	0.0204 (6)	0.0168 (6)	−0.0031 (5)	0.0029 (5)	−0.0024 (5)
C9	0.0172 (7)	0.0242 (6)	0.0235 (6)	0.0031 (4)	0.0054 (5)	0.0004 (5)
C10	0.0163 (7)	0.0237 (6)	0.0233 (6)	0.0012 (4)	0.0031 (5)	−0.0016 (5)
C11	0.0205 (7)	0.0353 (7)	0.0259 (7)	0.0040 (5)	0.0041 (5)	0.0060 (5)
C12	0.0165 (7)	0.0284 (6)	0.0271 (7)	0.0005 (5)	0.0041 (5)	0.0007 (5)

### Geometric parameters (Å, °)

**Table:**

O1—C2	1.2144 (14)	C6—C7	1.3936 (17)
O2—C2	1.3389 (15)	C7—C8	1.3913 (18)
O2—C1	1.4541 (14)	C7—H7	0.9500
O3—C5	1.3625 (13)	C8—H8	0.9500
O3—H3	0.8400	C9—C10	1.4855 (16)
O4—C6	1.3604 (14)	C9—H9A	0.9900
O4—C9	1.4394 (15)	C9—H9B	0.9900
C1—H1A	0.9800	C10—C12	1.4993 (17)
C1—H1B	0.9800	C10—C11	1.5043 (16)
C1—H1C	0.9800	C10—H10	1.0000
C2—C3	1.4816 (16)	C11—C12	1.4988 (17)
C3—C8	1.3845 (18)	C11—H11A	0.9900
C3—C4	1.4002 (16)	C11—H11B	0.9900
C4—C5	1.3792 (16)	C12—H12A	0.9900
C4—H4	0.9500	C12—H12B	0.9900
C5—C6	1.4028 (17)		
			
C2—O2—C1	116.08 (9)	C3—C8—C7	120.58 (11)
C5—O3—H3	109.5	C3—C8—H8	119.7
C6—O4—C9	118.13 (9)	C7—C8—H8	119.7
O2—C1—H1A	109.5	O4—C9—C10	108.28 (9)
O2—C1—H1B	109.5	O4—C9—H9A	110.0
H1A—C1—H1B	109.5	C10—C9—H9A	110.0
O2—C1—H1C	109.5	O4—C9—H9B	110.0
H1A—C1—H1C	109.5	C10—C9—H9B	110.0
H1B—C1—H1C	109.5	H9A—C9—H9B	108.4
O1—C2—O2	123.32 (11)	C9—C10—C12	117.02 (10)
O1—C2—C3	123.75 (12)	C9—C10—C11	120.08 (10)
O2—C2—C3	112.92 (10)	C12—C10—C11	59.87 (8)
C8—C3—C4	119.77 (11)	C9—C10—H10	116.0
C8—C3—C2	118.84 (10)	C12—C10—H10	116.0
C4—C3—C2	121.38 (12)	C11—C10—H10	116.0
C5—C4—C3	120.08 (12)	C12—C11—C10	59.90 (8)
C5—C4—H4	120.0	C12—C11—H11A	117.8
C3—C4—H4	120.0	C10—C11—H11A	117.8
O3—C5—C4	118.40 (11)	C12—C11—H11B	117.8
O3—C5—C6	121.48 (10)	C10—C11—H11B	117.8
C4—C5—C6	120.10 (10)	H11A—C11—H11B	114.9
O4—C6—C7	125.49 (11)	C11—C12—C10	60.23 (8)
O4—C6—C5	114.69 (10)	C11—C12—H12A	117.7
C7—C6—C5	119.81 (11)	C10—C12—H12A	117.7
C8—C7—C6	119.63 (12)	C11—C12—H12B	117.7
C8—C7—H7	120.2	C10—C12—H12B	117.7
C6—C7—H7	120.2	H12A—C12—H12B	114.9
			
C1—O2—C2—O1	−0.02 (15)	C4—C5—C6—O4	−177.57 (9)
C1—O2—C2—C3	−179.41 (9)	O3—C5—C6—C7	179.98 (10)
O1—C2—C3—C8	1.07 (16)	C4—C5—C6—C7	1.75 (15)
O2—C2—C3—C8	−179.54 (9)	O4—C6—C7—C8	178.38 (9)
O1—C2—C3—C4	179.92 (10)	C5—C6—C7—C8	−0.86 (16)
O2—C2—C3—C4	−0.69 (14)	C4—C3—C8—C7	0.76 (16)
C8—C3—C4—C5	0.13 (16)	C2—C3—C8—C7	179.63 (10)
C2—C3—C4—C5	−178.71 (9)	C6—C7—C8—C3	−0.39 (16)
C3—C4—C5—O3	−179.66 (9)	C6—O4—C9—C10	−174.90 (9)
C3—C4—C5—C6	−1.38 (15)	O4—C9—C10—C12	−168.23 (9)
C9—O4—C6—C7	−8.73 (15)	O4—C9—C10—C11	−99.07 (12)
C9—O4—C6—C5	170.55 (9)	C9—C10—C11—C12	−105.72 (12)
O3—C5—C6—O4	0.66 (14)	C9—C10—C12—C11	110.76 (12)

### Hydrogen-bond geometry (Å, °)

**Table:**

*D*—H···*A*	*D*—H	H···*A*	*D*···*A*	*D*—H···*A*
O3—H3···O1^i^	0.84	2.00	2.7808 (14)	153

Symmetry codes: (i) *x*−1/2, −*y*+1/2, *z*−1/2.
