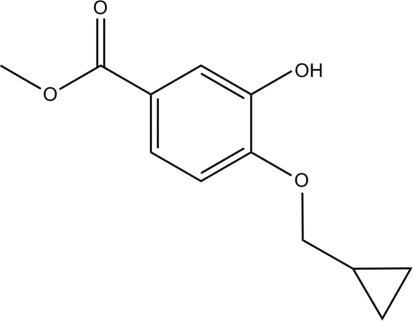# Methyl 3-(cyclo­propyl­meth­oxy)-4-hy­droxy­benzoate. Corrigendum

**DOI:** 10.1107/S1600536810050282

**Published:** 2010-12-18

**Authors:** Jing-Jing Hou, Xian-Chao Cheng, Run-Ling Wang, Shu-Qing Wang

**Affiliations:** a School of Pharmacy, Tianjin Medical University, Tianjin 300070, People’s Republic of China

## Abstract

The chemical diagram and the title of the paper by Hou *et al.* [*Acta Cryst.* (2010), E**66**, o2004] are corrected.

## Abstract 2

Corrigendum to *Acta Cryst.* (2010), E**66**, o2004.

In the paper by Hou *et al.* (2010) ▶, the chemical name given in the *Title* should be ‘Methyl 4-(cyclo­propyl­meth­oxy)-3-hy­droxy­benzoate’. The revised scheme is shown below.